# Giant Lumbar Disc Herniation Mimicking Spinal Tumor: A Case Report

**DOI:** 10.7759/cureus.96092

**Published:** 2025-11-04

**Authors:** Achraf Moussa, Hatim Belfquih, Manal Mernissi, Mohamed Abdellaoui, Ali Akhaddar

**Affiliations:** 1 Department of Neurosurgery, Avicenne Military Hospital of Marrakech, Cady Ayyad University, Marrakech, MAR; 2 Department of Radiology, Mohammed V Military Teaching Hospital, University of Mohammed V Souissi, Rabat, MAR; 3 Medical School, University of Mohammed V Souissi, Rabat, MAR

**Keywords:** epidural mass lesion, giant lumbar disc herniation, mri with contrast, paralyzing lumbosciatica, surgical excision

## Abstract

Giant lumbar disc herniation (GLDH) is common among adults aged 23-56 years who spend a significant amount of time seated or standing with heavy workloads. This case report discusses a 50-year-old male waiter with GLDH, leading to nerve root compression with neurological impairment. Initial magnetic resonance imaging (MRI) findings showed a differential diagnosis of GLDH and an epidural mass lesion. A follow-up MRI with contrast was performed to rule out more serious conditions, but it showed peripheral enhancement of the expansive process. The patient underwent surgical excision to alleviate symptoms and improve function. The diagnosis of GLDH can be difficult. The clinical presentation is hard to distinguish from other causes of lumbar canal stenosis, such as synovial cysts, epidural hematomas, metastases, and tumors.

## Introduction

Giant lumbar disc herniation (GLDH) is an unusual spinal condition characterized by the displacement of intervertebral disc material beyond its normal anatomical boundaries. They represent 8%-22% of all lumbar disc herniation. In some cases, the herniated fragment may become sequestered [1]. Disc sequestration refers to the migration of herniated intervertebral disc fragments into the epidural space. Due to the anatomical configuration of the anterior epidural space, these fragments typically migrate in a lateral, cephalad, or caudal direction.

This atypical migration pattern may mimic the radiological appearance of spinal neoplasms, potentially leading to misdiagnosis [2]. The pathological migration of disc material can result in significant pain and functional impairment due to nerve root compression. Clinical manifestations commonly include localized lower back pain and radiculopathy, often accompanied by sensory deficits such as numbness or weakness along the affected dermatome. In severe cases, signs of cauda equina syndrome (CES) may be present, including perineal hypoesthesia and bladder or bowel dysfunction.

Magnetic resonance imaging (MRI) with gadolinium contrast remains the key diagnostic modality for differential diagnosis. Management strategies range from conservative approaches such as physical therapy, and pharmacological treatment to more invasive interventions like epidural steroid injections or surgical decompression, depending on the severity of symptoms and response to initial treatment [3].

We present the case of a patient whose preoperative imaging raised suspicion of a spinal tumor, but whose final diagnosis of GLDH was confirmed by histological analysis.

## Case presentation

A 50-year-old man with no significant past medical history reported a one-month history of progressive symptoms that initially manifested as intermittent neurogenic claudication associated with mild weakness in the left lower limb. Shortly thereafter, low back pain developed, typically triggered by prolonged standing and heavy lifting. Over the subsequent weeks, the symptoms gradually intensified, with the onset of left-sided lumbosciatica accompanied by a progressive sensation of heaviness in the left lower limb.

On general physical examination, the patient was alert and oriented, with no signs of systemic illness or fever. Neurological assessment revealed a steppage gait on the left side. A lumbar spinal syndrome was present, along with radicular symptoms following the left L5 distribution. A motor deficit consistent with L5 involvement (foot dorsiflexion) was 1/5 (Medical Research Council scale (MRC)). There were no sensory deficits or genitourinary sphincter disturbances. Laboratory work-up was unremarkable.

**Figure 1:**
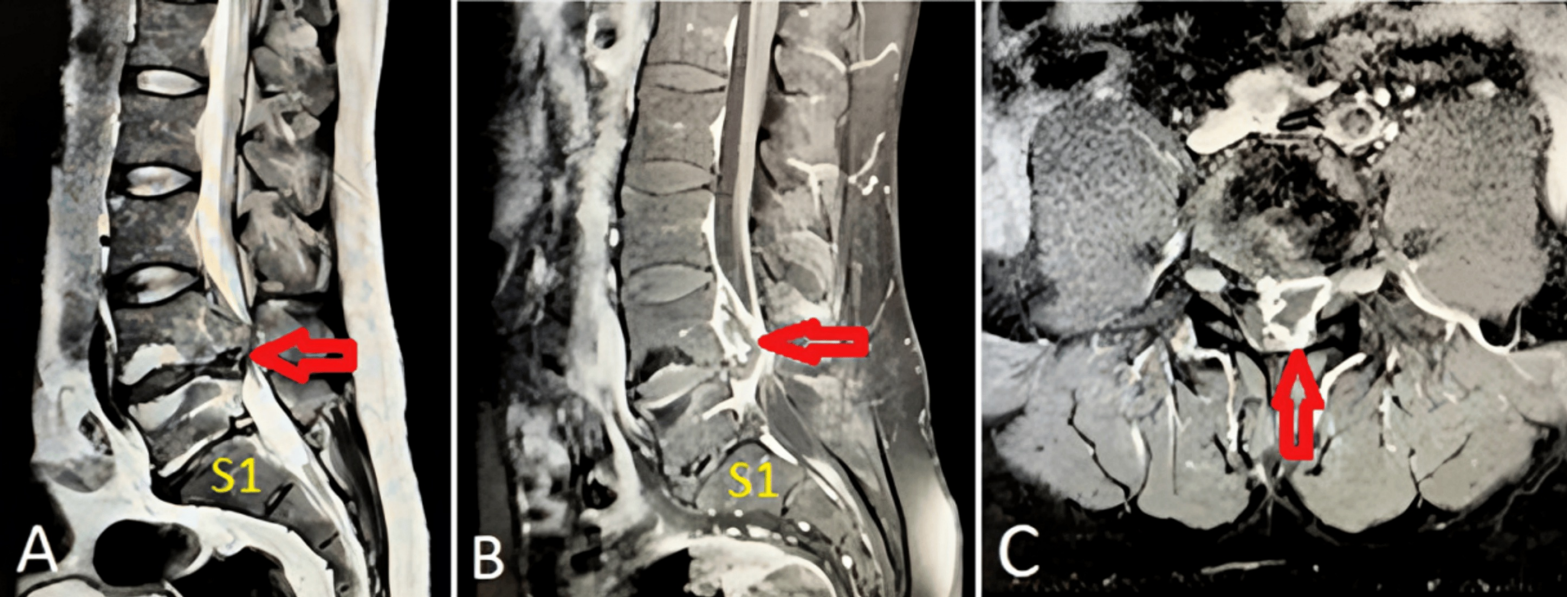
(A) Magnetic resonance imaging of lumbar spine in T2 sagittal without contrast identifies a symmetric epidural mass lesion (arrow) measuring 4x1.9x1.7 cm (CCxAPxT) at the epidural space of L4/L5. It shows T2W hyperintense signal and slight signal difference with the original disc. No bone erosion or destruction and the mass lesion compresses the L5 root in the left. The differential diagnosis includes epidural abscess and spinal tumor. (B and C) Magnetic resonance imaging of lumbar spine in T1 sagittal and axial with gadolinium contrast identifies that the L4/L5 epidural mass lesion shows with peripheral enhancement (arrow). CCxAPxT: Craniocaudal × Anteroposterior × Transverse

MRI lumbar spine showed a T2 heterogeneous epidural mass at the L4-L5 level, measuring 4.0×1.9×1.7 cm (craniocaudal×anteroposterior×transverse) and the lesion appears as hypointense with a core of hyperintensity (Figure 1A). A contrast-enhanced MRI with gadolinium revealed peripheral enhancement of the same lesion, confirming its epidural nature (Figure 1B, C).

**Figure 2:**
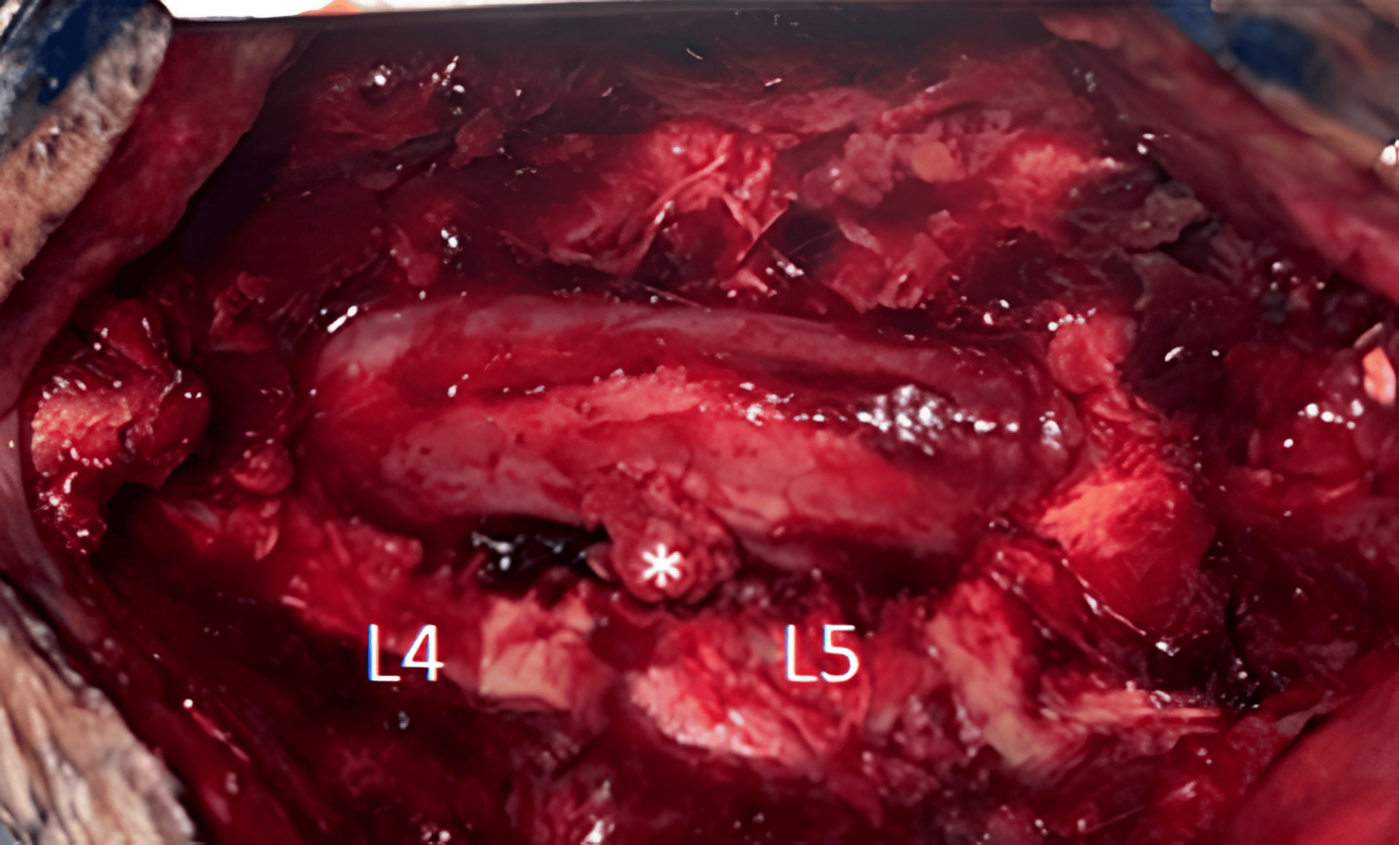
Intraoperative image showing a part of the expansive process similar to a herniated disc (Etoile).

**Figure 3:**
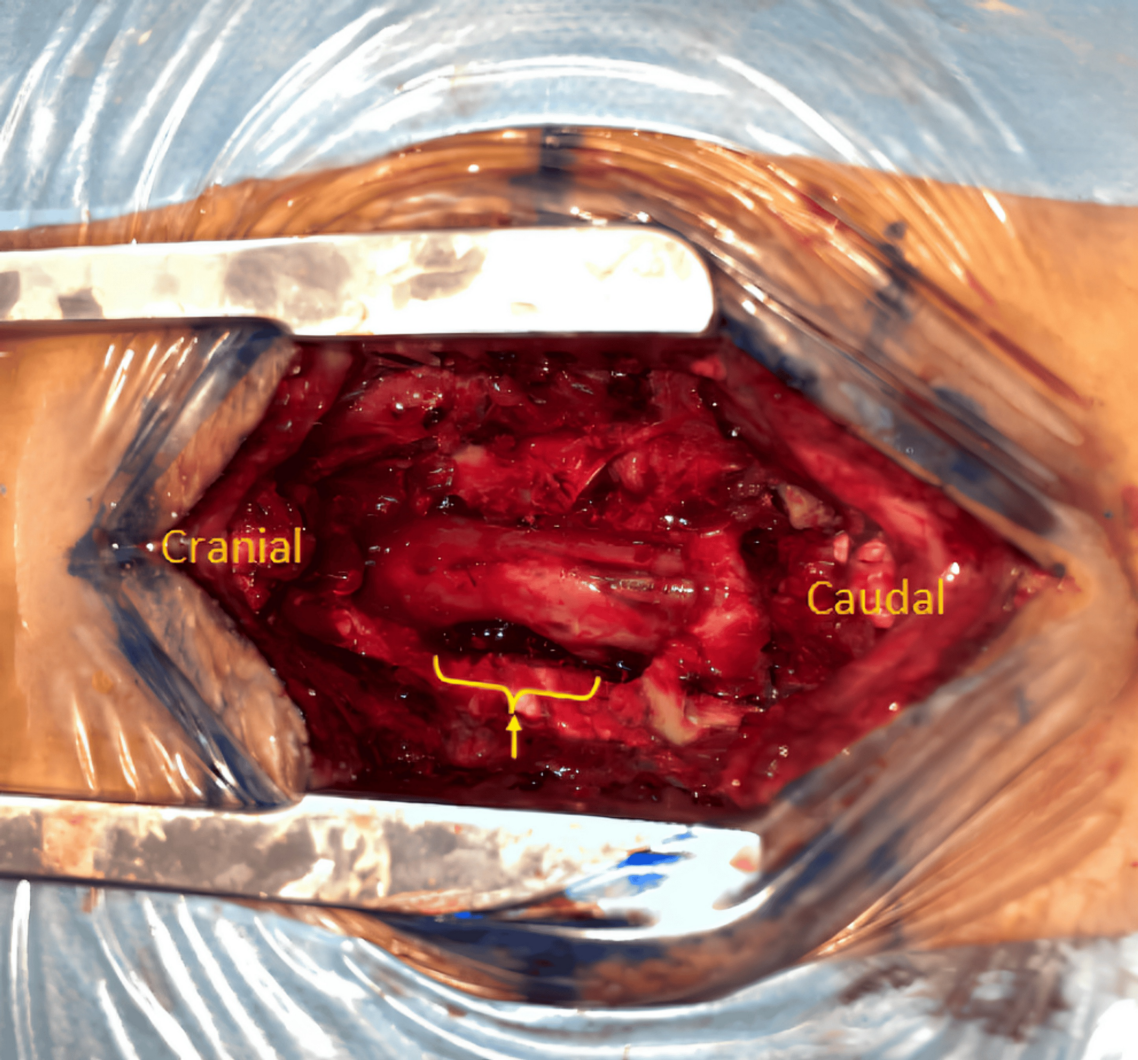
Intraoperative image showing the space created after the excision of the herniated disc. The arrow indicates the defect corresponding to the site of the removed lesion.

The patient underwent surgical decompression via L4-L5 laminectomy and left partial facetectomy. Intraoperatively, an epidural mass was identified occupying the anterior epidural space at the L4-L5 level. The lesion appeared consistent with a giant lumbar disc herniation, as indicated by the asterisk in (Figure 2). The mass was removed without performing a discectomy. The intervertebral disc space was noted to be narrowed, and an L5 foraminotomy was performed. At the end of the procedure, the L5 nerve root was free and decompressed (Figure 3).

**Figure 4:**
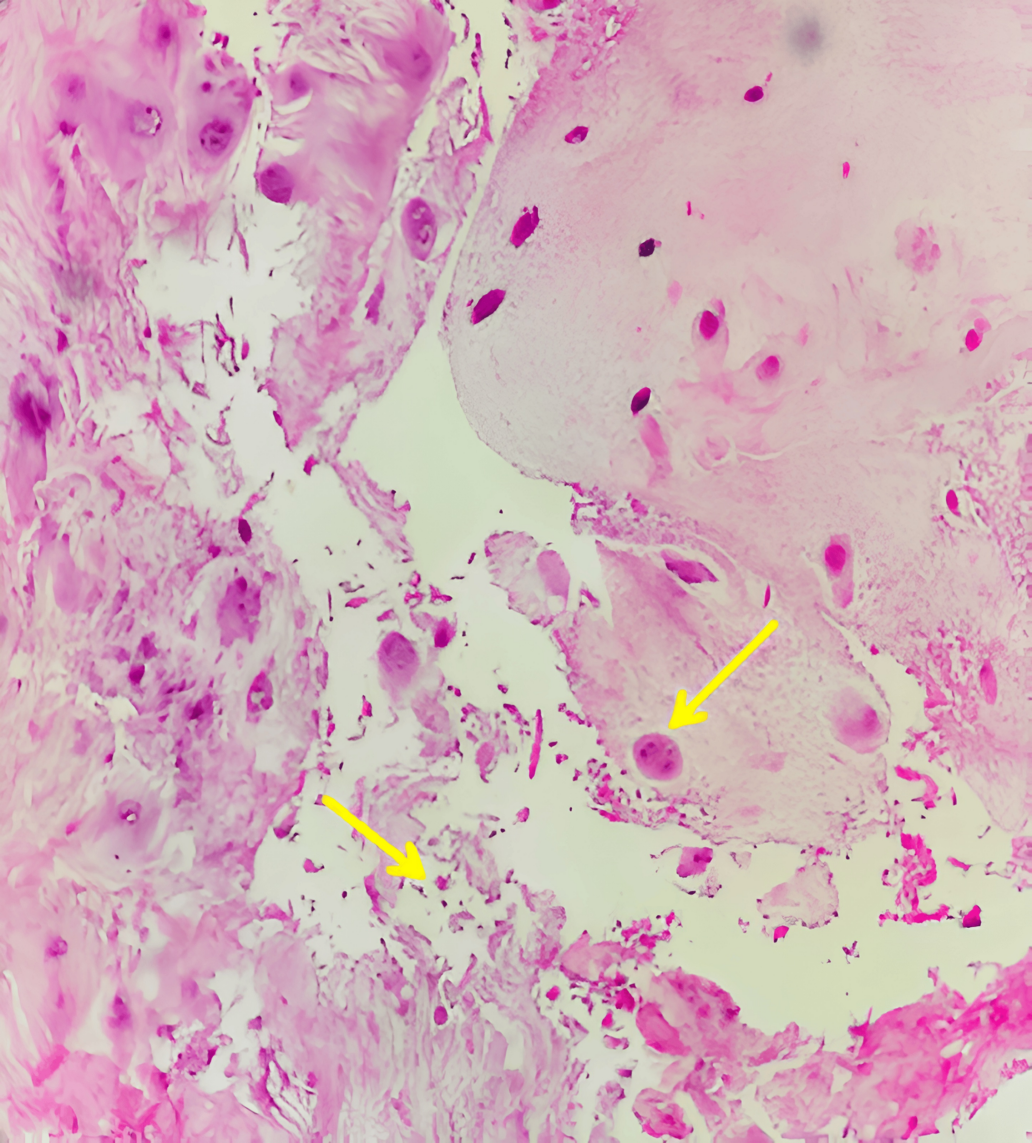
Microscopic image of disc material: chondrocytes proliferation, granular changes, mucous degeneration and structural alteration (arrow). (Hematoxylin-eosin stain, ×60).

Histopathological examination revealed a cartilaginous lesion (disc material) with no evidence of neoplastic proliferation (Figure 4). Postoperatively, the patient demonstrated a partial recovery of left lower limb motor function (MRC scale grade 3/5) following physiotherapy sessions, with complete resolution of lumbosciatica. At a three-month follow-up, the patient remained asymptomatic, with a residual L5 motor deficit.

## Discussion

GLDH is an uncommon variant of disc pathology, defined by the extrusion of disc material occupying more than 50% of the anteroposterior diameter of the lumbar spinal canal. Often referred to as "giant" or "large" disc herniations, these cases account for approximately 8% to 22% of all lumbar disc herniations. They predominantly affect the lower lumbar levels, with the L4-L5 and L5-S1 intervertebral discs being the most frequently involved [4].

The pathogenesis of GLDH is thought to be influenced by adhesions between the ventral dura mater and the posterior longitudinal ligament. These adhesions may be exacerbated by repeated microtrauma or prior spinal surgeries. Anatomically, the anterior epidural space is constrained by the posterior longitudinal ligament and the sagittal midline septum. This space, located between the vertebral body and the posterior longitudinal ligament, serves as a barrier that prevents herniated disc fragments from crossing the midline. Laterally, the membrane attaches to the posterior longitudinal ligament and extends to the lateral wall of the spinal canal, thereby limiting the posterior migration of free disc fragments [1]. Together, the posterior longitudinal ligament and lateral membrane function as anatomical constraints that direct the movement of herniated material. Consequently, most disc fragments tend to migrate in lateral, cranial, or caudal directions [5]. Posterior migration into the dorsal epidural space or intradural extension is rare. Although the lumbar region is the most frequently affected, cervical and thoracic intervertebral discs may also be involved in certain cases [6].

Patients with GLDH often present with severe unilateral or bilateral sciatica, which may be accompanied by varying degrees of neurological deficits. In more critical presentations, CES is observed in up to 20% of cases. Notably, among patients diagnosed with CES, approximately 45% to 60% have been found to have underlying giant lumbar disc herniation as the primary cause. Computed tomography (CT) is typically effective in identifying the herniated disc fragment, which generally exhibits a density similar to that of normal disc material. However, in some cases, particularly those involving centrally located, voluminous herniations, distinguishing the lesion from the dural sac can be challenging. Diagnostic accuracy can be enhanced by closely evaluating the surrounding epidural fat, which serves as an anatomical landmark. Additionally, bone window CT scans can aid in detecting associated degenerative changes of the spine, such as osteoarticular alterations secondary to chronic mechanical stress [3].

Although MRI is the modality of choice for detecting intraspinal soft tissue lesions, its specificity remains limited [6]. As such, it is essential to differentiate prolapsed intervertebral disc material from other epidural or intradural pathologies, including epidural abscesses, resolving epidural hematomas, synovial cysts, schwannomas (neurilemmomas), lipomas, and meningiomas. On MRI, free disc fragments within the spinal canal typically appear as hypointense on T1-weighted images and hyperintense on T2-weighted images. When contrast is administered, these fragments often demonstrate peripheral enhancement, which is a key feature distinguishing them from other enhancing spinal lesions [7].

Among the differential diagnoses considered, epidural abscesses typically occur in the posterior epidural space and demonstrate low to intermediate signal intensity on T1-weighted MRI and high signal intensity on T2-weighted sequences. Following contrast administration, they often exhibit homogeneous or peripheral rim enhancement. Subdural hematomas may present with similar imaging features; however, resolving epidural hematomas tend to appear as well-defined circular lesions with peripheral enhancement. These lesions often show signal intensities similar to cerebrospinal fluid (CSF), sometimes accompanied by “focal marks” on the spinal cord, which may represent adjacent cord compression or displacement. In contrast, the absence of enhancement on contrast-enhanced MRI is suggestive of a synovial cyst. Schwannomas (neurilemmomas) are generally located in the epidural space and typically appear isointense on T1-weighted images, hyperintense on T2-weighted images, and show intense contrast enhancement. Spinal lipomas are predominantly intradural, with over 50% found within the dura mater. They are characterized by high signal intensity on T1-weighted images, low signal intensity on T2-weighted images, and signal suppression on fat-saturated sequences, confirming their fatty composition. Meningiomas, which are most commonly located in the thoracic spine, are typically intradural in location. They appear isointense on both T1- and T2-weighted images and demonstrate uniform enhancement after contrast administration [8].

The standard treatment for GLDH involves early surgical decompression, typically performed via an interlaminar approach or laminectomy, depending on the size, location, and degree of migration of the herniated disc fragment. Several authors advocate for laminectomy in cases of giant or extensively migrated fragments, as it allows more effective decompression and facilitates safe disc removal through gentle retraction and discectomy [9].

In recent years, several surgeons have explored various minimally invasive surgical techniques for the management of GLDH, including minimally invasive transforaminal lumbar interbody fusion and percutaneous endoscopic lumbar discectomy. These approaches have demonstrated promising results, as they are associated with reduced surgical trauma, faster postoperative recovery, and favorable clinical outcomes [4].

Limited surgical exposure may necessitate excessive manipulation of neural elements during discectomy, increasing the risk of complications such as permanent neurological deficits, as previously reported by McLaren and Bailey [10]. Shapiro also advises against the use of microdiscectomy via hemilaminectomy for large disc herniations due to inadequate access and increased risk of injury [11].

In most cases, giant disc fragments are not adherent to the dural sac, allowing for relatively straightforward removal. However, when adhesions are present, careful and progressive dissection is critical to avoid dural tears or nerve root damage during fragment excision.

## Conclusions

GLDH can present a diagnostic challenge, particularly due to their potential to mimic spinal neoplasms on imaging. In this context, contrast-enhanced MRI plays a pivotal role in differentiating herniated disc material from other intraspinal pathologies. While certain cases of severe disc herniation, particularly those exhibiting high signal intensity on T2-weighted sequences, may respond favorably to conservative treatment modalities, such as lifestyle modifications, surgical decompression remains necessary when non-operative measures fail to alleviate symptoms or when there is neurological compromise involving nerve roots or the spinal cord. It is therefore imperative that clinicians, including chiropractors, integrate diagnostic imaging and clinical assessment effectively to formulate appropriate, individualized management plans for patients with GLDH.
